# Detergent-Assisted Protein Digestion—On the Way to Avoid the Key Bottleneck of Shotgun Bottom-Up Proteomics

**DOI:** 10.3390/ijms232213903

**Published:** 2022-11-11

**Authors:** Katerina Danko, Elena Lukasheva, Vladimir A. Zhukov, Viktor Zgoda, Andrej Frolov

**Affiliations:** 1 Department of Biochemistry, St. Petersburg State University, 199034 St. Petersburg, Russia; 2 All-Russia Research Institute for Agricultural Microbiology, Podbelsky Chaussee 3, Pushkin, 196608 St. Petersburg, Russia; 3 Institute of Biomedical Chemistry, 119121 Moscow, Russia; 4 K.A. Timiryazev Institute of Plant Physiology RAS, 127276 Moscow, Russia

**Keywords:** bottom-up proteomics, detergents, detergent-assisted proteolysis, filter-assisted sample preparation (FASP), gel-free proteomics, shotgun proteomics, sodium dodecyl sulfate (SDS), surfactants

## Abstract

Gel-free bottom-up shotgun proteomics is the principal methodological platform for the state-of-the-art proteome research. This methodology assumes quantitative isolation of the total protein fraction from a complex biological sample, its limited proteolysis with site-specific proteases, analysis of the resulted peptides with nanoscaled reversed-phase high-performance liquid chromatography-(tandem) mass spectrometry (nanoRP-HPLC-MS and MS/MS), protein identification by sequence database search and peptide-based quantitative analysis. The most critical steps of this workflow are protein reconstitution and digestion; therefore, detergents and chaotropic agents are strongly mandatory to ensure complete solubilization of complex protein isolates and to achieve accessibility of all protease cleavage sites. However, detergents are incompatible with both RP separation and electrospray ionization (ESI). Therefore, to make LC-MS analysis possible, several strategies were implemented in the shotgun proteomics workflow. These techniques rely either on enzymatic digestion in centrifugal filters with subsequent evacuation of the detergent, or employment of MS-compatible surfactants, which can be degraded upon the digestion. In this review we comprehensively address all currently available strategies for the detergent-assisted proteolysis in respect of their relative efficiency when applied to different biological matrices. We critically discuss the current progress and the further perspectives of these technologies in the context of its advances and gaps.

## 1. Introduction

The term “proteomics” was suggested by Marc Wilkins in 1996, who defined proteome as the “PROTein complement expressed by a genOME” [1]. In agreement with this, proteomics aims characterization of the entire proteome, including protein expression, post translational modifications (PTMs), cellular localization, and turnover aspects [2]. Due to the advances in mass spectrometry (MS) and highly efficient separation techniques, proteome research developed explosively over the recent decades [3]. Currently, the state-of-the-art equipment and technology give access to large-scale and high-throughput proteomics analysis [4], which might rely on the top-down and bottom-up strategies [5].

In terms of the top-down proteomics approach, both intact proteins and the products of their tandem mass spectrometric (MS/MS) fragmentation are directly analyzed by MS [6]. This is a powerful technique which gives access to nearly 100% sequence coverage and allows characterization of genetic variations of protein sequences, alternative splicing isoforms and proteoforms along with the related patterns of PTMs [7]. However, unfortunately, application of top-down proteomics is limited to the polypeptides with the molecular weights larger than 30 kDa [6], and requires high purities and quantities of the target proteins [1,2].

In contrast, bottom-up proteomics assumes limited enzymatic digestion of intact proteins (or protein mixtures) with subsequent analysis of resulted proteolytic peptides by MS and MS/MS [8,9]. This strategy is universal, independent of the size of digested proteins, and relies on detection of peptides, which are advantageous as analytes in comparison to proteins. Indeed, separation of peptides by reversed phase chromatography (RPC) is more efficient, as they are featured with higher ionization efficiency and yield predictable and informative fragmentation patterns [10]. Furthermore, in its state-of-the-art implementation, bottom-up proteomics makes analysis of complex biological matrices containing thousands of different proteins feasible. Therefore, this approach allows analysis of full proteomes in a broad range of biological matrices [11].

In general, the bottom-up proteomics employs two principal strategies which are referred to as gel-based and gel-free approaches. Most often, the gel-based approach relies on the separation of proteins by two-dimensional gel electrophoresis (2D-GE)—the off-line combination of isoelectric focusing (IEF) on immobilized pH gradients (IPG-strips) and polyacrylamide gel electrophoresis in the presence of sodium dodecyl sulfate (SDS-PAGE), followed by in-gel limited proteolytic digestion [12]. This technique, however, has several important limitations: (i) reproducibility of the 2D-GE strongly depends on stability of separation conditions [3]; (ii) 2D-GE is susceptible to technical errors manifested as a gel-to-gel discrepancy [13]; (iii) it lacks analytical resolution, i.e., fails to resolve the whole proteome as several proteins could co-migrate within one spot; and (iv) challenging in analysis of hydrophobic proteins due to their low abundance, difficulties of solubilization and precipitation during IEF [12].

In the case of the gel-free approach (often also referred to as shotgun or LC based proteomics), the protein mixture undergoes digestion with site-specific proteases prior to separation of the resulted proteolytic peptides [14,15,16]. Most often, this approach relies on the nanoscaled reversed-phase high performance (or ultra-high-performance) liquid chromatography (nanoRP-HPLC or -UHPLC) [17,18,19]. Due to its high efficiency, peak capacity, reproducibility, robustness, ease in practical implementation and flexibility of this method, this technique is widely used in most of the bottom-up proteomics applications [15]. NanoRP-HPLC is typically off-line combined with matrix-assisted laser desorption/ionization (MALDI) [20] or coupled on-line to electrospray ionization (ESI) [21].

However, the success of shotgun proteomics analysis is strongly dependent on the completeness of enzymatic digestion at the step of sample preparation [22]. The efficiency of proteolysis is mostly affected by the activity of proteases and accessibility of protein cleavage sites. Due to essential variations in solubility and aggregation potential of individual proteins in complex protein extracts, the latter might be a challenging task. Although detergents and chaotropic agents are absolutely mandatory for efficient digestion, their supplementation to protein solubilization buffers dramatically affects both separation and mass spectrometry-based detection of proteolytic peptides [3]. Indeed, detergents not only strongly interfere with RP-LC separation, but also disrupt ESI and suppress detection of peptides [23,24,25]. Therefore, multiple strategies employing degradable or removable detergents and methods using additional devices such as filters or beads were proposed recently to overcome these limitations [22,26,27]. Here, we present a comprehensive review of these techniques and their application in bottom-up gel-free proteomics. To cover this topic in the most comprehensive way, we employed a key word-based search against multiple public databases such as Scopus, PubMed, ScienceDirect, Web of Science, Semantic Scholar, etc., with a special emphasis on the factors affecting proteolysis efficiency and related effects on the output of the bottom-up proteomics experiment.

The primary search relied on the keywords “shotgun proteomics, detergents, acid-labile detergents” and was not limited by any time interval. At this step we found the absence of any comprehensive comparison of the different detergent-assisted workflows in the context of their implementation for various biological matrices. After planning the study and setting the main accents, we continued the search for each of the selected main points, i.e., generally independently for each chapter (so that the sets of keywords differed between the sections). Considering the review articles, we tried to include the most recent or highly cited publications. The time frame criterion was not strictly applied while conducting the search, although we avoided citing the works published before the last 20 years. Although, recent reports (published over the past five years) were addressed in the highest priority, the earlier ones still were not ignored. Considering different methods of sample preparation (the main stress of the work), strongly predominant are the original articles were included. The set of keywords typically included the following terms: (i) MS-based proteomics strategy (bottom-up shotgun proteomics, gel-based or gel-free proteomics); (ii) description of proteomics workflow (in-solution digestion, in-gel digestion); (iii) name of the protease (trypsin, LysC etc.); (iv) name of the detergent and its type (SDS, SDC, ionic or non-ionic detergents, acid-labile surfactants etc.); (v) the name or the description of detergent removal strategy (filter-aided sample preparation, S-Trap, acidification etc.); and (vi) biological matrix (provided as name of organism or as cell/tissue/organ type).

## 2. Proteases Used for Gel-Free Proteomics

Limited proteolysis represents the critical step in any bottom-up proteomics workflow, which can be accomplished by an array of available site-specific proteases [28]. Among them, trypsin is one the most widely used, and tryptic digestion has become the golden standard for enzymatic proteolysis in bottom-up proteomics [29]. Trypsin is a highly specific and efficient serine protease that cleaves proteins at the carboxyl side of arginine or lysine residues. As tryptic digestion yields proteolytic peptides with C-terminal lysine or arginine residues, this protease is well-compatible with RPC separation methods, available peptide fragmentation techniques, and search algorithm-based identification protocols [28]. Importantly, autolysis of trypsin during the proteolysis leads to the formation of pseudotrypsin which possesses chymotryptic activity. This activity results in non-specific cleavages at C-terminus of aromatic amino acid residues, negatively affecting, thereby enzyme specificity and suppressing principal trypsin activity [30]. Therefore, commercially available trypsin products are modified and featured with suppressed autolytic activity. For example, stabilization of the enzyme can be achieved by the modification of trypsin cleavage site by selective methylation of the specific lysine residue [31].

However, the completeness of the enzymatic digestion can be essentially influenced by the protein sequence. Thus, trypsin activity can be suppressed if lysine or arginine residues are: (i) flanked by acidic amino acid residues [32]; (ii) neighbored by phosphorylated serine and threonine [33]; or (iii) followed by proline [34]. It was also reported that efficiency of proteolysis is higher at the arginine cleavage sites in comparison to the lysine ones [35]. This fact could be explained by differential affinity of these two residues to the active site of the enzyme [36]. The success of proteolysis also depends on the nature of the protein substrate, presence and topography of disulfide bonds, secondary and tertiary structures, as well as the patterns of post-translational modifications [37].

However, it needs to be considered that hydrophilic globular lysine- and arginine-rich proteins yield relatively short proteolytic peptides, i.e., approximately 56% of the generated peptides are not exceeding six amino acid residues [38]. Thus, some of these peptides might lack hydrophobic amino acid residues and suffer from compromised retention on the reversed phase and can be, therefore, lost during the nanoLC-MS/MS analysis [39]. Moreover, such short protein tag sequences make distinguishing between closely related protein isoforms and identification of various PTMs extremely difficult, if not virtually impossible [40]. This would ultimately result in a gap in the sequence information on the corresponding segment of the proteome [41].

The compromised sequence coverage, protein identification and PTM detection rates might be improved by employment of alternative proteases, such as chymotrypsin, LysC, LysN, ArgC, GluC, LysargiNase (Table 1) [40]. These enzymes were shown to be applied either individually [42,43,44] or in a combination with other proteases (mainly with trypsin) [41,45,46,47,48]. For example, Guo et al. demonstrated that consecutive digestion of HeLa cell lysates with multiple proteases significantly increased the protein identification rates and sequence coverage [49].

Interestingly, GluC was used for characterization of protein glycation patterns [42]. On the other hand, LysN was successfully applied to the study of N-terminal modifications of proteins [44], whereas ArgC proved to be well suited for analysis of C-terminal sequences [50]. In contrast to trypsin, chymotrypsin cleaves proteins at the carboxylic side of hydrophobic amino acid residues and yields, therefore, orthogonal (in respect to tryptic digestion) patterns of proteolytic peptides. Importantly, this digestion specificity might give access to highly hydrophobic membrane regions [51,52].

However, despite the numerous surveys demonstrating the advantages of using alternative proteases or multi-enzyme proteolysis strategies, trypsin remains the protease of choice for LC-based proteomics studies. It could be illustrated by the fact that tryptic peptides account for the vast majority (>90%) of peptides from data depository Global Proteome Machine Database, whereas the literature search clearly indicates that alternative proteases are rarely applied in recently developed digestion strategies [45,53,54]. Limited employment of alternative proteases in gel-free bottom-up proteomics could be attributed to several reasons. On one hand, it can be explained by a broad variation in the optimal conditions (pH, temperature, concentration of unfolding reagents) of the enzymatic reactions catalyzed by each protease [40]. On the other, the proteolytic peptides generated by alternative proteases might have more or less compromised ionization and fragmentation characteristics leading to less confidence of peptide identification [55]. It should be noted that multiple enzyme digestion strategy is usually time consuming, requires higher amounts of sample, and complicates further analysis of complex proteolytic mixtures, especially when false discovery rate (FDR) correction applied for all searches together [56].

**Table 1:** Alternative proteases employed in shotgun proteomics.

Protease	Family	Cleavage Site	Application	Reference
LysC	Serine protease	C-terminal of K	Used in combination with trypsin to improve digestion efficiency	[57]
GluC	Serine protease	C-terminal of E (at pH 4) C-terminal of D (at pH 8)	Analysis of glycated proteins	[42]
Chymotrypsin	Serine protease	C-terminal of F, Y, L, W and M	Analysis of proteins transmembrane regions	[51,52]
LysN	Metalloprotease	N-terminal of K	Analysis of N-terminal modifications	[44]
AspN	Metalloprotease	N-terminal of D	Used in combination with other enzymes to improve digestion efficiency	[49,58]
ArgC	Cysteine protease	C-terminal of R	Used in combination with other enzymes to improve digestion efficiency and analyze C-termini of proteins	[47,50]

## 3. Detergents

**Figure 1:**
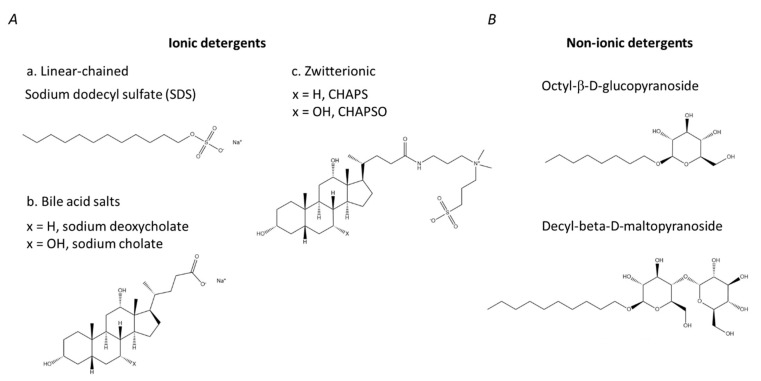
Structures of different classes of detergents. (**A**)—ionic detergents; (**B**)—non-ionic detergents.

Detergents and/or chaotropes are essential for protein digestion as they destroy protein secondary and tertiary structure and facilitate, thereby, the access of proteases to their specific cleavage sites [59]. Detergents are often supplemented to lysis buffers, i.e., used to facilitate sample disruption in parallel to protein solubilization and denaturation [60]. One needs to take into account, however, that for the samples of low complexity and single/few cell approaches freeze/thawing cycles are found to be sufficient for sample lysis [60], and detergents can be employed downstream in the protocol when quantitative extraction of membrane proteins is required [61]. Detergents (often also referred to as surfactants [62]) are amphipathic organic molecules ultimately containing a hydrophobic long-chain aliphatic nonpolar tail and a hydrophilic polar head group [63] (Figure 1). By their structural organization, these compounds are analogous to phospholipids which are the major constituents of cellular membranes [64]. Thus, detergents mimic the natural protein environment and facilitate, thereby, solubilization of membrane proteins. Due to this similarity in structural organization, the hydrophobic tails of detergents readily penetrate the phospholipid bilayer, and the cell membranes can be destroyed via repulsing of their polar heads [65,66,67]. In the aqueous solution, detergents form spherical structures—micelles, in which hydrophobic tails of molecules form the interior core and hydrophilic head groups are exposed to the aqueous solvent [62].

Based on their chemical characteristics, all detergents are categorized into two groups: ionic (anionic, cationic or zwitterionic) and non-ionic. Ionic detergents contain a hydrophobic hydrocarbon chain or steroidal backbone, and a charged head group which can be either anionic or cationic (Figure 1A). Linear-chained ionic detergents, such as sodium dodecyl sulfate (SDS), possess strong solubilization properties, i.e., can break protein-lipid, protein–protein and lipid–lipid complexes and are considered, therefore, to be harsh denaturants. Bile salts are ionic detergents containing steroidal backbone. Their derivatives—sodium deoxycholate (SDC) and 3-[3-(cholamidopropyl) dimethyl-ammonio]-1-propanesulfonate (CHAPS), are less strong detergents with less pronounced denaturing properties in comparison to linear chain detergents [68].

Non-ionic detergents have uncharged hydrophilic head groups (Figure 1B). Due to this structural feature, these compounds typically disrupt only protein–lipid and lipid–lipid interactions, whereas proteins remain non-denatured [69]. Therefore, these agents are usually defined as mild non-denaturing surfactants. Accordingly, application of mild detergents allows extraction of membrane proteins in their native state, without disruption of the protein–protein interactions [70]. Moreover, non-ionic detergents are commonly used for isolation and purification of lipid rafts [71]. One needs to keep in mind, however, that the majority of the available non-ionic detergents are UV-active and interfere with spectrophotometric methods of protein determination, which are typically employ detection at 280 nm [72].

Zwitterionic detergents combine the properties of ionic and non-ionic detergents (Figure 1A). The structure of zwitterionic detergents assumes the presence of hydrophilic zwitterionic head groups, which contain both cationic and anionic parts and possess, therefore, zero net charge. Such surfactants have a lower denaturing potential than ionic detergents but are harsher than non-ionic detergents as they are able to break protein–protein interactions [65].

Finally, the chaotropic agents disrupt weak non-covalent interactions (hydrogen bonding, dipole–dipole and hydrophobic interactions), thereby facilitating protein denaturation, which usually remains reversible. These compounds stabilize the unfolded state of proteins by electrostatic interactions and hydrogen bonding [73]. Urea is commonly applied as a nonionic chaotropic agent, used in sample preparation workflows either individually or in a combination with thiourea or/and detergents. However, application of urea for protein solubilization has some limitations. Thus, since proteolytic enzymes may be also denaturated by urea, high concentrations of this agent may lead to inhibition of their specific activities. It should be kept in mind that in aqueous solution a small proportion of urea is converted to ammonium cyanate, which readily carbamylates proteins [74]. Although heating of protein samples could facilitate protein denaturation, the temperature above 30 °C may lead to thermal degradation of urea accompanied with formation of isocyanic acid. This by-product is directly involved in carbamylation at N-termini or side chains of lysine residues, and therefore might inhibit protein cleavage by proteases [73].

Despite the absolute necessity of using detergents and chaotropes in sample preparation workflows for mass spectrometry-based proteomics analysis, these chemical agents should be removed prior to nanoLC-MS, as they are incompatible with both RPC separation and ionization by ESI. Indeed, the detergents like Triton X-100, Tween, or NP-40 contain polyethylene glycol (PEG), which is retained on the reversed phase and elutes throughout the whole LC gradient and interferes with both analyte retention and detection [10]. Even at the levels as low as 0.01% (*w*/*v*), SDS is capable of completely suppressing signals of peptide ions [23,24,25]. Moreover, detergents inhibit evaporation of eluents in the droplets formed during the ESI process and impede the transfer of analytes into the gas phase, which dramatically affects the sensitivity of the whole analysis [3]. Therefore, as application of detergents in proteomics workflows cannot be avoided, their removal prior to LC-MS analysis becomes the critical step of sample preparation and one of the most difficult bottlenecks dramatically, affecting efficiency of proteolysis.

Despite of this critical role of detergents, highly efficient protein denaturation can be achieved with other approaches. For example, in-gel digestion represents one of the most widely used techniques to ensure practically quantitative proteolysis at all potential cleavage sites in proteins. On the other hand, this technique, employing 1D-GE for protein separation, represents an efficient method for the removal of detergents, chaotropes and other contaminants that may interfere with the subsequent LC-MS analysis [75]. However, it should be noted that a higher amount of protein (and protease, consequently) is required compared to the in-solution digestion approach [76]. Moreover, quantitative analysis is less accurate in the case of in-gel digestion strategy because of unpredictable sample losses. Therefore, in-gel digestion is a laborious, less reproducible, and cost-inefficient method in comparison to the in-solution digestion techniques. Although this strategy is well suited for the analysis of specific protein fractions, a gel-free approach remains the main workhorse used for high-throughput untargeted and unbiased shotgun proteomic analysis [3].

## 4. Strategies for the Removal of Detergents during Sample Preparation

In agreement with this, numerous detergent removal strategies were successfully established for a broad range of commercially available surfactants with confirmed efficiency of protein solubilization and digestion. Due to its outstanding potential for solubilization of proteins, SDS is currently recognized as one of the strongest available detergents, being the most widely used ionic surfactant [77,78]. Although this detergent is incompatible with LC-MS experiments per se, multiple methods for its efficient removal after protein delipidation and denaturation are to date introduced in routine proteomics practice (Section 4.1).

An alternative approach to avoid the disadvantages associated with the application of detergents relies on degradable surfactants, which are compatible with LC-MS analysis—PPS Silent, ProteaseMAX, RapiGest, AALS II, SDC, which are addressed in more detail in Section 4.2.

### 4.1. Methods for Removal of SDS

One of the earliest methods for SDS removal was suggested by Botelho and colleagues and relied on SDS precipitation in acetone (Table A1) [79]. Unfortunately, this approach appeared to be time consuming and suffered from low throughput accompanied with high sample losses, which were essential for hydrophobic (e.g., membrane-associated) proteins.

Recently, Zhou et al. showed that SDS-assisted digestion followed by SDS precipitation with potassium chloride (KCl) to form insoluble potassium dodecyl sulfate (KDS) allowed efficient and approximately quantitative SDS removal (>99.9%) [80]. This approach demonstrated much better proteome coverage, which was comparable with the state-of-the-art detergent assisted proteolysis. Surprisingly, in terms of the membrane protein identification rates, the KDS-based SDS removal appeared to be superior in comparison to alternative surfactants (RapiGest), chaotropic agents (urea) and organic solvents (methanol). However, this method requires careful adjustment of the KCl amounts supplemented to analyzed samples. Indeed, the concentration of the working KCl is critically important for the efficiency of SDS removal and might affect recovery of proteolytic peptides and LC-MS analysis [81].

The method proposed by Sun and colleagues relied on strong cation exchange (SCX) chromatography and was free from this limitation. This protocol ensured high recovery rates for proteolytic peptides and high efficiency of SDS removal. The authors particularly noted that this technique could be easily incorporated in LC-based proteomics strategy as it was successfully employed as the first dimension of an integrated LC × LC-MS/MS approach [82].

Despite the numerous existing strategies for SDS removal, during the recent decade the filter-aided sample preparation (FASP) method was successfully introduced and proved to be the most efficient approach, allowing purification of the sample from SDS and chemicals interfering with proteolysis [54,78,83,84,85]. This technique is based on the use of centrifugal spin columns containing membrane filters of specific molecular-weight cut-off (typically 30 kDa) and acting as one-way reactors for digestion. After the completion of digestion, the proteolytic peptides could be eluted by the centrifugal force, while high-molecular weight compounds (e.g., polysaccharides and nucleic acids) remained in the filter unit.

Unfortunately, the first version of this method proposed by Manza and co-workers [86] suffered from essential sample losses and incomplete removal of SDS. Further, Wisniewski et al. modified this method by introducing urea in the protocol for efficient (≥99.9%) depletion of the detergent. According to this approach, after protein solubilization in SDS and loading the resulted solution in the filter unit, the detergent was replaced with a urea solution in a series of multiple washing steps [78]. Remarkably, FASP also represents an efficient approach for removal of the alkylation and reducing agents used in digestion, as well as sample matrix components—nucleic acids, polysaccharides and lipids which might interfere with downstream analysis [75]. The Mann laboratory also introduced multiple enzyme digestion (MED)-FASP protocol enabling the sequential application of two or three enzymes for efficient proteolysis [87]. This method extension resulted in dramatic improvement of sequence coverage, increasing protein identification rates and numbers of identified modification sites.

Another modification of FASP, employing 0.2% (*w*/*v*) SDC supplemented to the exchange, alkylation, and digestion buffers, is usually referred to as enhanced FASP (eFASP) [83]. Implementation of SDC into proteomic workflows was proposed to enhance protein digestion and increase sequence coverage [88]. Further improvement of the method performance (up to three-fold reduction of the sample losses) can be achieved by the passivation of the filter unit membranes with 5% (*w*/*v*) Tween-20 prior to the experiment. The performance of FASP and eFASP was evaluated in terms of the number of identified proteins and overall proteome coverage. These two techniques showed no significant differences at the level of protein identification, although they differed slightly in the physicochemical properties of the corresponding proteolytic peptides. Thus, FASP and eFASP can be treated as complementary when analyzing complex mixtures of proteolytic peptides [89].

Although FASP was originally proposed for digestion of animal cell and tissue lysates, this technique is currently widely employed in shotgun proteomics of various plant organs—leaves [90,91,92], seeds [93], roots [94], fruits [95] and flowers [96] (Table A1). Thus, FASP can be treated as a robust and reproducible method for both animal [54] and plant [97] proteomics.

Suspension trapping (S-trap) method, which is based on the similar principle as FASP, was proposed by Zougman et al. in 2014 [98]. S-trap packed filters consist of two parts: a quartz or borosilicate depth filter and a reversed-phase C8 membrane. An acidified protein solution in SDS (pH < 1) is mixed with a methanol solution with neutral pH and applied to the filter. The protein suspension is trapped in the quartz filter, and SDS is dissolved in the methanol and washed away along with salts and other components interfering with LC-MS. The proteins are then hydrolyzed with an appropriate protease and the resulting peptides are desalted and concentrated in a reversed-phase C8 membrane for further HPLC-MS analysis. Comparative study revealed that S-trap outperformed the FASP method by the number of unique protein and peptide identifications [99].

Single-pot solid-phase-enhanced sample preparation (SP3) proteomics workflow, proposed by Hughes et al., represents a distinctive strategy giving access to the rapid, efficient and high-throughput sample preparation for shotgun proteomics [100]. This method employs carboxylate-coated paramagnetic beads with hydrophilic surface, which could efficiently bind to proteins and peptides in the presence of organic solvent in a manner analogous to hydrophilic interaction chromatography (HILIC) or electrostatic repulsion hydrophilic interaction chromatography (ERLIC). While proteins and peptides become immobilized on the beads, chemical contaminants (such as detergents, chaotropes, salts) could be removed from the solution through several rapid rinsing steps. Importantly, this method eliminates the need for peptide desalting, so that the eluted peptides could be directly subjected to MS analysis. Overall, SP3 method demonstrates high reproducibility when handling with sub-microgram amounts of starting material (<10 μg) and allows highly sensitive analyses with just minimal sample losses [101]. Further improvements of this method aimed at improvement of protein recoveries through the optimization of the strategies for cell/tissue lysis, binding, rinsing and elution conditions [102,103,104]. The SP3 strategy was successfully applied for animal- [103,105,106] and plant-derived matrices [107].

It is necessary to note that despite their efficiency, all the mentioned methods of SDS removal suffer from essential losses, accompanying sample preparation. In this context, special attention needs to be paid to selective losses, which might cause alterations in proteome profile, whereas proportional losses (affecting all proteins in the mixture at the same degree) can be considered as acceptable. Thus, the workflows for SDS removal need to be appropriately adjusted to a specific sample matrix and/or target proteome part to minimize proportional sample losses and to avoid the selective ones [82].

It is important to note that removal of SDS is not necessary when enzymatic digestion is followed by depletion or enrichment procedures. This can be exemplified by the workflows established in our group for proteomics analysis of protein glycation sites in human blood plasma [108] and plant tissues [109], which comprise enrichment of glycated tryptic peptides by boronic acid affinity chromatography (BAC) after tryptic digestion with supplementation of 10% (*w*/*v*) SDS to the solubilization buffer. We have unambiguously demonstrated that both at the qualitative [108] and quantitative [110,111] level no effect on the method performance could be observed, i.e., SDS supplemented at the step of tryptic digestion could be quantitatively removed during the subsequent BAC procedure.

### 4.2. Detergents Compatible with LC-MS

**Figure 2:**
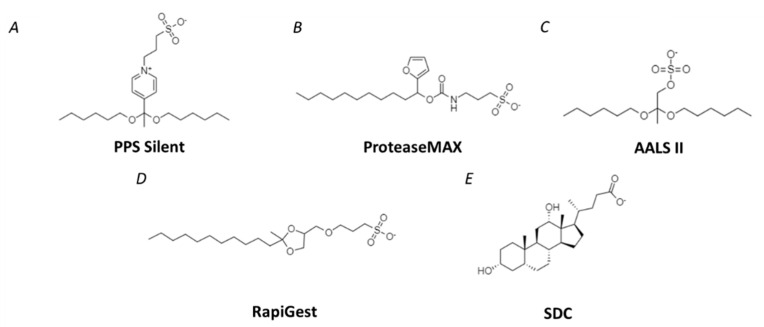
Structure of degradable and removable detergents applied in shotgun bottom-up proteomics. (**A**–**D**)—acid-labile surfactants, (**E**)—detergent forming insoluble precipitates.

Over the last decade, several commercially available MS-compatible detergents were successfully introduced in the everyday practice. The majority of the available MS-friendly surfactants can be attributed to one of the two principal groups: (i) acid-labile surfactants (with PPS Silent, ProteaseMAX, AALS II, RapiGest as the most widely spread representatives) containing acid-labile functional groups which can be hydrolyzed at acidic pH or/and upon heating; and (ii) detergents yielding insoluble precipitates under low pH (e.g., SDC). Accordingly, the resulting degradation products or precipitates can be removed by solid phase extraction (SPE) or centrifugation, respectively. The structures of MS-compatible detergents are represented in Figure 2.

The degradable detergents available by different vendors vary in their structures. Thus, PPS Silent (3-[3-(1,1-bisalkyloxyethyl)pyridin-1-yl]propane-1-sulfonate, (Figure 2A) is a zwitterionic acid cleavable surfactant that rapidly decomposes at low pH into products which do not possess any surfactant properties and do not interfere with downstream MS analysis. Any precipitates formed upon supplementation of the PPS Silent solution are, most likely, not related to the protein component of the sample and can be easily removed by centrifugation [112]. PPS Silent was successfully applied to a broad range of biological matrices—animal cells and tissues [113], bacteria [114] and plasma [115]. However, today it is only minimally employed in plant proteomics [116] (Table A1).

ProteaseMAX™ Surfactant (sulfonate sodium 3-((1-(furan-2-yl)undecyloxy)carbonylamino) propane-1-sulfonate)) is a degradable hydrophobic anionic acid-labile detergent (Figure 2B) [117]. In contrast to PPS Silent, this surfactant, when applied in the concentrations of 0.1–0.2% (*w*/*v*), can be hydrolyzed simultaneously with enzymatic proteolysis. Thus, a separate step of detergent degradation is not necessary anymore in the digestion protocol and can be, therefore, omitted. However, even in the case of incomplete degradation of the detergent (that can be the fact when shorter degradation times applied or the reagent concentration of is too high), ProteaseMAX Surfactant can be degraded either in 0.5% (*v*/*v*) TFA for 15 min at 37 °C, or at neutral pH by incubation at 95 °C for five minutes. Acidic hydrolysis of the surfactant yields two products—hydrophilic zwitterionic 3-aminopropane-1-sulfonic acid and neutral hydrophobic 1-(furan-2-yl)undecan-1-ol [117]. Hydrophilic product can be easily removed by RP-SPE, while the hydrophobic product is poorly soluble in water and centrifugation might be necessary for its quantitative removal [118]. To date, ProteaseMAX was successfully applied to the proteomics studies of animal or human cells [119], tissues [120,121,122] and plasma [45,122]. On the other hand, it was promising in the proteomics analysis of potato (*Solanum tuberosum*) and tomato (*Phaseolus vulgaris*) leaf [123,124] (Table A1). However, ProteaseMAX was shown to introduce artifactual modifications on cysteine residues of proteins. These modifications have high physical and chemical resemblance with S-palmitoylation and hydroxyfarnesylation. Therefore, this detergent should be used with caution in bottom-up proteomics workflows, especially when analyzing modifications of proteins with lipids or with the products of their oxidative degradation [117].

AALS II (Anionic acid labile surfactant II, Progenta) represents one of the most widely used acid-labile detergents (Figure 2C) [125]. Implementation of this agent in routine digestion protocols gives access to quantitative protein solubilization, improvement of cell lysis protocols, optimization of enzymatic digestion, and reducing material losses due to surface adsorption via non-specific interactions [126]. As AALS II is the structural analog of SDS [125], its protein solubilization potential is close to that of SDS [127]. However, in contrast to SDS, it can be hydrolyzed upon the proteolysis by adjusting pH to 2.5–3 with 1% (*v*/*v*) trifluoroacetic acid (TFA) and subsequent treatment for 10 min at 37 °C. The resulted hydrophilic degradation products do not show any surfactant activity, minimally interact with sample matrix and analytes, and are shown to not interfere with MS [125,126] (Table A1).

Importantly, other anionic, cationic and zwitterionic detergents such as AALS I, cationic acid labile surfactant I/II (CALS I/II) and zwitterionic acid labile surfactant I/II (ZALS I/II) are available by Progenta. Thereby, AALS II, CALS II, and ZALS II are featured with a higher protein solubilization potential, i.e., they are more suitable for reconstitution of hydrophobic proteins and are especially efficient in membrane proteomics [3]. This detergent was successfully optimized for the digestion of protein isolates obtained from seeds of *Brassica napus* [128] and *Pisum sativum* L. [129,130] as well as legume nodules [131]. AALS II was also used to study the phosphorylation of retinal tissue receptors [132], mammalian cell culture [133], blood serum [53] (Table A1).

RapiGest, (3-[(2-methyl-2-undecyl-1,3-dioxolan-4-yl) methoxy]-1-propanesulfo- nate), is an acid-cleavable anionic detergent used to facilitate the enzymatic digestion of proteins (Figure 2D). As other detergents, RapiGest efficiently enhances the availability of protein cleavage sites for all standard proteases used in proteomics (trypsin, Lys-C, Asp-N and Glu-C) and improves, thereby, the efficiency of protein digestion [134]. Upon the digestion, RapiGest can be hydrolyzed in the presence of 0.5% (*v*/*v*) TFA (45 min at 37 °C). The cleavage reaction yields two degradation products—2-dodecanone and 3-(2,3-dihydroxypropyloxy)-1-propanesulfonic acid sodium salt. While the former product is insoluble in water and can be efficiently removed from the proteolytic mixture by centrifugation, the latter one is water soluble and can be quantitatively removed by RP-SPE [135]. This detergent is actively used in shotgun proteomics of animal and human cells, tissues and plasma [136,137,138]. Recently, it was also employed in plant proteomics—for example, a comprehensive study of barley seed protein isolates [139] (Table A1).

SDC is an ionic bile salt surfactant which is found in the in the gastrointestinal tract and impacts on solubilization of lipid-related nutrients and facilitates, thereby, their digestion [140]. Its structure comprises a planar steroid moiety and a short aliphatic side-chain (Figure 2E). Due to this structural organization, in contrast to the other above considered detergents, SDC has polar and non-polar faces, but a not well-defined tail and polar head groups [141]. SDC is a relatively mild detergent and affects enzymatic activities to a lesser extent in comparison to linear-chain detergents with the same head group [65]. Under acidic conditions this detergent readily forms insoluble deoxycholic acid precipitate, which can be easily removed by centrifugation [142]. Therefore, SDC is well-compatible with LC-MS/MS-based shotgun proteomics and is widely used in proteomics workflows. However, it was shown that a substantial proportion of unique peptides might co-precipitate with SDC that might be associated with sample losses and decrease in protein sequence coverage [143].

Therefore, recently, Masuda et al. proposed the phase-transfer (PTS) method as an alternative for SDC removal [88]. After addition of a water-immiscible organic solvent (e.g., ethyl acetate) to the aqueous tryptic hydrolysate, SDC can be quantitatively extracted into the organic phase, while the SDC-free aqueous phase can be used for the further analysis [88]. Interestingly, it was also shown that SDC is capable of increasing trypsin activity up to five-fold when used in concentrations from 0.01 to 1% (*w*/*v*) [88]. To date, SDC is widely and successfully applied for plasma proteomics [144,145]. Lin et al. showed that SDC is capable of membrane protein solubilization when used in high concentrations (5%) [146]. The SDC-based method was also demonstrated to be less time- and work-consuming in comparison to filter-aided approaches and could be applied for efficient proteomics analysis of plant samples—barley leaves [90] and oil palm [147] (Table A1).

## 5. Comparison of Different Protein Digestion Strategies in Terms of Their Efficiency

The state-of-the-art proteomics relies on a broad panel of sample preparation, pre-fractionation, enrichment and depletion methods, which give access to efficient protein identification and quantification in practically all animal, plant, bacterial and fungal objects. As numerous detergents with widely varying properties are available at the market, their potential for protein solubilization needs to be characterized and compared in terms of their performance with different matrices. This would allow choosing the appropriate digestion strategy and the most efficient protein solubilization agents (first of all, detergents), which would be the most suitable to address the objectives of each specific research. Therefore, this aspect was comprehensively addressed during the recent decade.

Thus, Waas et al. compared MS-compatible detergents such as Invitrosol, ProteaseMAX, RapiGest, PPS Silent Surfactant and AALS I/II [148]. The pellets prepared from the membranes of STO mouse embryonic fibroblasts were solubilized with these detergents. The authors showed that, in terms of the number of identified proteins, peptides, and sequence coverage, AALS I and II appeared to be the most favorable detergents for investigation of membrane proteins [148]. Further, Pirmoradian and co-workers compared three protein solubilization strategies employing SDC, ProteaseMAX and urea for shotgun proteomics analysis of A375 cell line. It was demonstrated that ProteaseMAX outperformed SDC- and urea-based methods in terms of the number of identifications and sequence coverage [119] (Table A1).

Wu and coworkers also studied the potential of several detergents (PPS Silent, RapiGest and SDC) for solubilization of membrane proteins of *Escherichia coli* and mammalian cell line MCF7. The experiments included solubilization of membrane protein fraction with 1% (*w*/*v*) of RapiGest, 1% (*w*/*v*) PPS Silent or 1% (*w*/*v*) SDS. It should be noticed that only RapiGest mixture was boiled at 100 °C for 5 min in order to enhance protein denaturation (as required by the vendor instructions). To remove the detergent, the SDS-containing samples were pre-cleaned by SCX chromatography. Although the authors claimed that RapiGest had the highest power in characterization of membrane proteome [135], the uncertainty with the results of protein determination and the lack in description of the corresponding methodology makes interpretation of the quantitative results difficult. Moreover, the overperformance of RapiGest, observed by the authors, can be explained by the difference in the sample preparation workflows. Indeed, for example, SDS removal by SCX might be associated with considerable losses of proteolytic peptides [149].

Other research presented by Porter et al. compared the efficiency of SDC, PPS Silent, Invitrosol and RapiGest with protein extracts from 293 kidney cell lines (HEK293). Proteins were solubilized in 0.1% (*w*/*v*) RapiGest, 0.1% (*w*/*v*) Invitrosol, 0.1% (*w*/*v*) PPS Silent and 1% (*w*/*v*) of SDC. Surprisingly, no significant difference between individual solubilization methods and agents could be observed. Although the protein solubilization potential of RapiGest was superior in comparison to that of SDC, the numbers of the identified peptides were higher in the samples treated by SDC [150]. This observation can be explained by suppression of trypsin activity in the presence of RapiGest and/or by co-precipitation of hydrophobic peptides with the products of RapiGest cleavage [151].

In the study by Baniasad et al. performed on skin tissue samples, the performance of SDC was compared with several MS compatible detergents, namely RapiGest, PPS Silent, Invitrosol and ProteaseMAX. The study revealed higher peptide and protein identification rates for RapiGest-treated samples in comparison to those processed in the presence of ProteaseMAX when the same digestion conditions were applied. However, the authors highlighted that SDC turned out to be a much more cost-efficient LC-MS-compatible reagent which performed similarly to other more expensive detergents [152].

A comparison of sample preparation protocols for the bottom-up proteomics analysis of the secretome isolated from the islets of Langerhans was recently proposed by Schmudlach et al. [153]: the authors’ three FASP-based protocols, which relied on three different detergents—RapiGest, NP-40 and SDS. It was shown that application of SDS yielded the best quality spectra, the highest peptide and protein identification rates, and the larger number of hits for extracellular and vesicular proteins.

In turn, Chen and co-workers compared application of MS-compatible surfactants (RapiGest, PPS Silent, Invitrosol) for global analysis of the mammalian brain proteome. To increase the solubility of hydrophobic proteins, the authors used concentrated solutions of the detergents (1% (*w*/*v*) RapiGest and PPS Silent, 5X stock Invitrosol). Among the three detergents RapiGest allowed identification of the highest number of transmembrane proteins [26].

Despite the impressive progress in sample preparation techniques, LC-MS instrumentation developments and data processing pipelines, in terms of efficiency of the standardized workflows and numbers of high-quality publications, plant proteomics still considerably falls behind animal proteome research [154]. This fact can be explained by several objective challenges. On one hand, due to recalcitrant cell walls and high abundance of polymers and metabolites strongly interfering with extraction procedures (polysaccharides, terpenes, phenolics, organic acids, and pigments), protein isolation from plant tissues is challenging [32,91]. On the other, high dynamic range in the tissue contents of individual proteins efficiently prevents identification of low-abundant polypeptides by the data-dependent acquisition (DDA) algorithm. Finally, proteomics investigation of plants is strongly limited by relatively few available complete genome sequences of plant species and hence a lack of fasta files for sequences databases search [154].

However, several studies were accomplished to find the most efficient detergent for plant proteomics. For example, comparison of MS-compatible detergents (PPS Silent, RapiGest), a chaotropic reagent (guanidine hydrochloride), and an organic solvent (methanol) for the study of microsomal proteins in tomato roots revealed that application of RapiGest yielded the highest peptide identification rates, indicating that this protein solubilization agent had the strongest denaturing potential among the compared digest additives. On the other hand, it was shown that guanidine hydrochloride and methanol selectively enhanced detection of hydrophobic proteins. Thus, the integral approach including both detergent- and guanidine hydrochloride/methanol-based digestion strategies might be more efficient in investigation of membrane proteomes and membrane-rich proteome fraction [155] (Table A1).

Introduction of FASP in the routine practice of plant biology essentially enhanced the potential of plant shotgun proteomics. Thus, the conventional standard FASP protocol was found to outperform the SDC-based “in-solution” digestion method when used for shotgun proteomics analysis of barley leaves [90]. It was shown that standard FASP and SDC-FASP protocols performed in a similar manner. However, SDC-FASP method was featured with more efficient detection of highly hydrophobic proteins compared to classical FASP [90].

## 6. Conclusions

Currently, bottom-up gel-free shotgun proteomics is the one of the most widely spread MS-based platforms of post-genomic research. It relies on limited proteolysis of complex protein isolates, nanoLC-MS and MS/MS analysis of resulted mixtures of proteolytic peptides, and data analysis based on sequence database search and relative or absolute quantification. Among these three principal steps of the shotgun proteomics workflow, in solution proteolytic digestion still remains the principal bottleneck, critically affecting the overall output of the proteomics experiment. The major challenge thereby is to ensure efficient solubilization and denaturation of all proteins, which is a pre-requisite for exhaustive and reproducible cleavage of all protease-specific sites in all polypeptides constituting the complex protein isolate. Obviously, incomplete accessibility of the cleavage sites for proteases ultimately results in reduced peptide identification rates, less numbers of annotated proteins and, hence, compromised proteome coverage which cannot be repaired by employment of alternative proteases or multi-enzyme digestion approach.

In this context, application of detergents and chaotropes is absolutely mandatory to achieve complete degradation of the protein secondary and tertiary structure and, thereby, to ensure the access of proteases to their specific cleavage sites. However, these chemical agents are mostly incompatible with both RP-HPLC and ESI-MS and dramatically affect peptide separation and ionization. Therefore, multiple filter-aided and filter-free digestion strategies employing a broad range of degradable or/and removable surfactants are currently employed in shotgun proteomics. Thereby, filter-aided methods rely on digestion in membrane filter units with subsequent centrifugal removal of the detergent, whereas filter-free strategies assume post-digestion removal of the detergents or application of MS-compatible surfactants which could be degraded under specific conditions (pH, temperature).

Unfortunately, despite numerous coexisting protein solubilization/digestion strategies, the knowledge about their relative efficiency, as well as the underlying factors and mechanisms, is still limited. The comparative studies are relatively few, typically relying on individual selected matrices that are strongly biased for mammalian/human origin. Moreover, these matrices are typically quite easy to process (cell cultures, sub-cellular fractions, blood plasma), whereas the studies addressing complex organs (like total brain proteome profiling survey from Musunuri et al. [156]) can be considered as an exclusion from this tendency. The situation with plant proteomics is even worse. Indeed, the most complex plant matrices—green leaf, fruits, and seeds, remain only barely addressed, which can probably be explained by an essential gap in sample preparation methods in this field.

Therefore, the next step would be a comprehensive comparison of detergent-assisted digestion strategies, both across a broad panel of surfactants/chaotropes and sample matrices, widely varying in origin and complexity. Certainly, organelle proteomics and multi-staged LC-LC and LC × LC workflows need to be addressed as well. This approach will clearly demonstrate which digestion strategy is more suitable for a specific matrix, compartment, and multi-staged workflow.

## Data Availability

Not applicable.

## Appendix A

**Table A1:** Sample preparation strategies used in bottom-up proteomics.

#	Type of Sample	Methodology							
		Lysis/Protein Isolation	Detergent or Chaotrope	Reduction/ Alkylation	Protease	Detergent Removal Strategy	Chromatographic System	MS	Reference
1	*S. cerevisiae* lysate (insoluble fraction)	Ultrasound	SDS	+/+	trypsin	Pierce detergent removal spin columns	RP, C18 Phenomenex, water-ACN grad., 0.1% (*v*/*v*) FA	ESI-LIT-MS	[77]
	*C. elegans* lysate (soluble fraction)	Mechanically							
	Human embryonic kidney cell line (HEK293T)	Freeze/thaw							
2	HeLa cell line, BSA	Freeze/thaw, sonication	SDS, urea	DTT/IAA	(1) trypsin (2) LysC (3) ArgC (4) GluC (5) AspN (6) Chymotrypsin	FASP	RP, C18, 5–40% MeCN in 0.5% (*v*/*v*) acetic acid	ESI-LIT (LTQ)-Orbitrap-MS	[78]
	Mouse brain tissue	Mechanically, freeze/thaw, ultrasound							
	Mouse liver tissue	Mechanical, freeze/thaw, ultrasound							
3	Yeast	(1) French press 20,000 psi, GELFrEE Frac (2) Acetone precipitation (3) Chloroform/methanol-water precipitation	SDS	none	trypsin	FASP	RP, C18 Phenomenex	ESI-LIT(LTQ)- MS	[79]
4	*Shewanella oneidensis* MR-1	Barocycler and pressure 35,000 psi	SDS, Urea	DTT/IAA	trypsin	(1) FASP (2) KDS precipitation	RP, C18 Phenomenex	ESI-LIT-MS	[80]
	Mouse brain tissue	Mechanically							
	Mouse liver tissue	Mechanically							
5	Rat liver membrane enriched fraction	Mechanically, centrifugation, sucrose density fractionation	(1) SDS (2) Urea (3) RapiGest	DTT/IAA	trypsin	(1) FASP (2) KDS precipitation	RP, C18, PepMap, water-ACN grad., 0.1% (*v*/*v*) FA	ESI-HCIT-MS	[81]
6	*Escherichia coli* membrane fraction	Cell disruptor at 25,000 psi	(1) SDS (2) RapiGest (3) PPS Silent	DTT/IAA	trypsin	SCXX	RP, C18, Atlantis, water-ACN grad., 0.1% (*v*/*v*) FA	ESI-QqTOF-MS	[82]
	Breast cancer cell line (MCF-7) membrane fraction	Triton X-114-based lysis buffer, centrifugation							
7	*Escherichia coli*	Sonication	(1) DCA/ N-lauroyl sarcosine (2) SDS	(1) DTT/IAA (2) DTT/4-VP (3) TCEP/IAA (4) TCEP/4-VP	trypsin	FASP	RP, C18, water-ACN grad., 0.1% (*v*/*v*) FA	ESI-QqTOF HDMS or ESI-QqTOF-MS	[83]
8	Normal human cholangiocyte (H69) cell line		SDS, CHAPS Urea	DTT/IAA	trypsin	FASP	RP, C18, water-ACN grad., 0.1% (*v*/*v*) FA	ESI-TripleTOF (QqTOF-MS)	[84]
9	*Mytilus galloprovincialis* hepatopancreas	Sonication	SDS	(1) none (2) DTT/IAA	(1) trypsin (2) trypsin/Lys-C	(1) FASP (2) SP3 (3) S-Trap	RP, C18, water-ACN grad., 0.1% (*v*/*v*) FA	ESI-Q-Exactive HF (Q-Orbitrap-MS)	[85]
	*Scophthalmus maximus* liver								
10	HEK293 cell line	CelLytic NuCLEAR Extraction kit	SDS	TCEP+DTT/IAA	trypsin	FASP	RP, C18, water-ACN grad., 0.1% (*v*/*v*) FA	ESI-LIT(LCQ)-MS	[86]
	BSA								
11	HeLa cell line	Freeze/thaw, sonication	SDS	IAA	(1) GluC (2) ArgC (3) LysC (4) AspN (5) trypsin (6) chymotrypsin	FASP	RP, C18 ReproSil-Pur, water-ACN grad., 0.1% (*v*/*v*) acetic acid	ESI-LIT(LTQ)-Orbitrap-MS	[87]
	Mouse brain tissue	Mechanically, sonication							
	Mouse liver tissue	Mechanically, sonication							
12	HeLa cell line membrane enriched fraction	Mechanically	(1) RapiGest (2) SDC	DTT/IAA	LysC/trypsin	(1) SDC: acid precipitation (2) SDC: phase transfer surfactant	RP, C18 ReproSil-Pur, water-ACN grad., 0.1% (*v*/*v*) acetic acid	ESI-LIT(LTQ)-Orbitrap MS or QSTAR QqTOF-MS	[88]
	*Escherichia coli* membrane enriched fraction	Sonication	(1) RapiGest (2) SDC	DTT/IAA	LysC/trypsin	(1) SDC: acidprecipitation (2) SDC: phase transfer surfactant	C18 ReproSil-Pur, water-ACN grad., 0.1% (*v*/*v*) acetic acid	ESI-LTQ-Orbitrap MS or QSTAR QqTOF-MS	[88]
13	*Escherichia coli*	Freeze/thaw	(1) SDS (2) DCA	DTT/IAA	trypsin	FASP	RP, C18 Luna, water-ACN grad., 0.1% (*v*/*v*) FA	ESI-Q-Exactive (Q-Orbitrap-MS)	[89]
14	*Hordeum vulgare* L. cv. Golden promise (barley) leaves	Mechanically, sonication	(1) SDS (2) SDC (3) SDC/CAA	IAA	trypsin	FASP	RP, C18 ReproSil-Pur, water-ACN grad., 0.1% (*v*/*v*) FA	ESI-Q-Exactive (Q-Orbitrap-MS)	[90]
15	*Arabidopsis thaliana* shoots, roots, seeds	(1) Chloroform/methanol-water precipitation (2) Phenol extraction (3) Urea-based extraction (4) SDS-based extraction	(1) Urea (2) SDS	TCEP/IAA	LysC/trypsin	FASP	RP, C18, water-ACN grad., 0.1% (*v*/*v*) FA	ESI-Q-Exactive HF (Q-Orbitrap-MS)	[91]
16	*Ramonda serbica* (Phenix plant)	(1) TCA/acetone precipitation (2) Phenol-based extraction (3) Detergents-based extraction	(1) SDS (2) Triton X-100 (3) dodecyl-β-D-maltoside	DTT/IAA	trypsin	FASP	RP, C18, water-ACN grad., 0.1% (*v*/*v*) FA	ESI-LIT(LTQ)-Orbitrap-MS	[92]
17	*Triticum aestivum* L. (wheat) roots	TCA/acetone precipitation	SDS	IAA	trypsin	FASP	RP, C18, water-ACN grad., 0.1% (*v*/*v*) FA	ESI-Q-Exactive (Q-Orbitrap-MS)	[94]
18	*S. lycopersicum* (tomato) fruits	Sonication	SDS	DTT/IAA	trypsin	FASP	RP, C18, water-ACN grad., 0.1% (*v*/*v*) FA	ESI-Q-Exactive (Q-Orbitrap-MS)	[95]
19	*Ziziphus jujuba* Mill. flowers	TCA/acetone precipitation	SDS	DTT/IAA	trypsin	FASP	RP, C18, water-ACN grad., 0.1% (*v*/*v*) FA	ESI-Q-Exactive (Q-Orbitrap-MS)	[96]
20	Human prostate cancer (PC-3) cell line	(1) Phenol-based extraction (2) Detergent-based extraction	SDS, urea	DTT/IAA	trypsin	FASP	RP, C18, PepMap, water-ACN grad., 0.1% (*v*/*v*) FA	ESI-LIT(LTQ)-Orbitrap-MS	[97]
	*Arabidopsis thaliana* shoots								
	*Pisum sativum* (pea) seeds	(1) Phenol-based extraction (2) Detergent-based extraction	SDS, urea	DTT/IAA	trypsin	FASP	RP, C18, PepMap, water-ACN grad., 0.1% (*v*/*v*) FA	ESI-LIT(LTQ)-Orbitrap-MS	[97]
21	HeLa cell line, membrane enriched fraction	Sonication	(1) SDS (2) Triton X-114	DTT/IAA	(1) trypsin (2) trypsin/LysC	S-Trap	RP, C18, water-ACN grad., 0.1% (*v*/*v*) FA	ESI-LIT(LTQ)-Orbitrap-MS	[98]
22	Colorectal cancer (SW480) cell line	Sonication	Urea, SDS	DTT/IAA	trypsin	(1) FASP (2) S-Trap	RP, C18, water-ACN grad., 0.1% (*v*/*v*) FA	ESI-Q-Exactive (Q-Orbitrap-MS)	[99]
23	Human blood plasma	none	SDS	TCEP/IAA	trypsin	Chromatography	RP, C18, water-ACN grad., 0.1% (*v*/*v*) FA	ESI-LIT(LTQ)-Orbitrap-MS	[108]
24	Human blood plasma	none	SDS	TCEP/IAA	trypsin	Boronic acid affinity chromatography	RP, C18, water-ACN grad., 0.1% (*v*/*v*) FA	ESI-LIT(LTQ)-Orbitrap-MS	[111]
25	Mouse kidney tissue	Microdissection	PPS Silent	DTT/CAA	trypsin	degradation under acidic conditions	RP, C18	ESI-LIT(LTQ)-Orbitrap-MS	[113]
	Mouse pancreas/pancreatic islets	Sonication							
26	*Salmonella enterica* outer membrane vesicles	LPI™ FlowCells	PPS Silent	none	trypsin	degradation under acidic conditions	RP, C18 ReproSil-Pur, water-ACN grad., 0.1% (*v*/*v*) FA	ESI-LIT(LTQ)-FT-MS	[114]
27	*Arabidopsis thaliana* shoots	(1) SDS/Phenol-based extraction (2) Methanol/Chloroform extraction	PPS Silent	none (in-gel digest)	trypsin	degradation under acidic conditions	RP, C18 ReproSil-Pur, water-ACN grad., 0.1% (*v*/*v*) FA	ESI-QqTOF-MS	[116]
28	Insect Sf9 cell line, His-tagged RGS4	Sonication, CHAPS	ProteaseMAX	DTT/IAA	trypsin	degradation under acidic conditions	RP, C18 ReproSil-Pur, water-ACN grad., 0.1% (*v*/*v*) FA	MALDI-TOF/TOF or ESI-LIT(LTQ)-Orbitrap-MS	[117]
29	Mouse liver tissue	Mechanically, Extraction kit	ProteaseMAX	DTT/IAA	LysC/trypsin	degradation under acidic conditions	RP, C18 Easy-Spray, water-ACN grad., 0.1% (*v*/*v*) FA	ESI-LIT(LTQ)-Orbitrap-MS	[120]
30	Mouse brain tissue	Liquid microjunction (LMJ) technique	ProteaseMAX	DTT	trypsin	heating	PLRP-S, water-ACN grad	MALDI-MSI and ESI-Q-Exactive (Q-Orbitrap-MS)	[121]
31	Human glioblastoma tissue (whole tissue lysate, cytosol, microsomes, and plasma membrane fractions)	Mechanically	Urea, ProteaseMAX	DTT/IAA	(1) trypsin (2) LysC/trypsin	degradation under acidic conditions	RP, C18 ReproSil-Pur, water-ACN grad., 0.1% (*v*/*v*) FA	ESI-TripleTOF QqTOF-MS	[122]
	Human blood plasma								
32	PLRV-infected *Solanum tuberosum* (potato) leaves	Beads-assisted extraction	ProteaseMAX	TCEP/methanethiosulfonate	trypsin	none	RP, C18 ReproSil-Pur, water-ACN grad., 0.1% (*v*/*v*) FA	ESI-LIT(LTQ)-Orbitrap-MS	[123]
33	*Phaseolus vulgaris* L. (running bean) leaves	Methanol/acetone	Urea, ProteaseMAX	DTT/IAA	trypsin	none	RP, C18 Easy-Spray, water-ACN grad., 0.1% (*v*/*v*) FA	ESI-LIT(LTQ)-Orbitrap-MS	[124]
34	Mixture of myoglobin, BSA, B-casein, and ovalbumin	none	(1) SDS (2) ALSI	none (in-gel digest)	trypsin	degradation under acidic conditions	none	MALDI-TOF-MS	[127]
35	*Arabidopsis thaliana*	Phenol-based extraction	Urea, thiourea, AALS II	TCEP/IAA	trypsin	degradation under acidic conditions	RP, C18 PepMap, water-ACN grad., 0.1% (*v*/*v*) FA	ESI-LIT-Q-Orbitrap-MS	[128]
36	*Pisum sativum* (pea) seeds	Phenol-based extraction	Urea, thiourea, AALS II	TCEP/IAA	trypsin	degradation under acidic conditions	RP, C18 PepMap, water-ACN grad., 0.1% (*v*/*v*) FA	ESI-Q-Orbitrap-MS	[129]
37	*Pisum sativum* (pea) seeds	Phenol-based extraction	Urea, thiourea, AALS II	TCEP/IAA	trypsin	degradation under acidic conditions	RP, C18 PepMap, water-ACN grad., 0.1% (*v*/*v*) FA	ESI-LIT-Q-Orbitrap-MS	[130]
38	*Phaseolus vulgaris* L. (running bean) nodules	Phenol-based extraction	AALS II	TCEP/IAA	trypsin	degradation under acidic conditions	RP, C18, water-ACN grad., 0.1% (*v*/*v*) FA	ESI-LTQ-Orbitrap-ETD-MS	[131]
39	Mouse eye retina	Sonication	AALS II	TCEP/IAA	trypsin	degradation under acidic conditions	nanoUPLC	ESI-QqTOF-MS	[132]
40	MDCK II Tet-off cell line	Chloroform/methanol extraction	AALS II	DTT/IAA (in-gel digest)	trypsin	degradation under acidic conditions	RP, PicoFrit, water-ACN grad., 0.1% (*v*/*v*) FA	ESI-LIT(LTQ)-Orbitrap-MS	[133]
41	*Escherichia coli* membrane fraction	French press 20,000 psi	(1) SDS (2) RapiGest (3) PPS	DTT/IAA	trypsin	(1) SDS: SCX (2) RapiGest and PPS Silent: degradation under acidic conditions	RP, C18, water-ACN grad., 0.1% (*v*/*v*) FA	ESI-QqTOF-MS	[135]
	Human breast cancer (MCF7) cell line membrane fraction	French press 20,000 psi, aceton							
42	Human blood plasma	none	RapiGest	DTT/IAA	trypsin	degradation under acidic conditions	RP, C18, water-ACN grad., 0.1% (*v*/*v*) FA	ESI-QqTOF-MS	[136]
43	U-2 OS osteosarcoma cell line	Sonication	RapiGest	TCEP/IAA	trypsin	degradation under acidic conditions	RP, C18, water-ACN grad., 0.1% (*v*/*v*) FA	ESI-LIT(LTQ)-Orbitrap MS	[137]
	U251 glioblastoma cell line								
	HeLa CCL2 cell line								
	Human (BT-474) breast cancer cell line								
	Jurkat cell line								
	Murine pre-adipocytes								
44	Rat ligodendro- cytes, oligodendrocyte precursor cells	Sonication, chloroform/methanol extraction	(1) SDS (2) RapiGest	DTT/acrylamide	trypsin	FASP	RP, C18 ReploSil-Pur, water-ACN grad., 0.1% (*v*/*v*) FA	ESI-LIT(LTQ)-Orbitrap MS	[138]
45	*Hordeum vulgare* L (barley) caryopse	TCA/acetone precipitation	RapiGest	DTT/IAA	trypsin	degradation under acidic conditions	RP, C18, water-ACN grad., 0.1% (*v*/*v*) FA	ESI-QqTOF-MS	[139]
46	Rat liver membrane enriched fraction	Mechanically, centrifugation, sucrose density fractionation	SDC	DTT/IAA	trypsin	(1) acid precipitation (2) phase transfer surfactant	RP, C18 PepMap, water-ACN grad., 0.1% (*v*/*v*) FA	ESI-3D-HCIT-MS	[142]
47	Human blood plasma	none	SDC	DTT/IAA	trypsin	acid precipitation	RP, C18 PepMap, water-ACN grad., 0.1% (*v*/*v*) FA	ESI-LIT(LTQ)-FT-MS	[143]
48	Human blood plasma	none	SDS	TCEP/IAA	trypsin	trap column	RP, C18 Aquity, water-ACN grad., 0.1% (*v*/*v*) FA	ESI-LIT-Q-Orbitrap-MS	[144]
49	Human blood plasma	none	SDS	TCEP/IAA	trypsin	acid precipitation	RP, C18, water-ACN grad., 0.1% (*v*/*v*) FA	ESI-QqLIT-MS	[145]
50	Rat liver enriched membrane fraction	Sonication	(1) SDS (2) SDC	DTT/IAA	trypsin	(1) SDC: acid precipitation (2) SDS: FASP	RP, C18 PepMap, water-ACN grad., 0.1% (*v*/*v*) FA	ESI-3D-HC-LIT-MS	[146]
51	*Elaeis guineensis* (oil palm) fruit mesocarps	TFA/acetone precipitation	Urea, thiourea, CHAPS, SDC	TCEP/IAA	trypsin	SDC: acid precipitation	RP, C18 PepMap, water-ACN grad., 0.1% (*v*/*v*) FA	ESI-Q-Exactive Orbitrap-MS	[147]
52	Mouse embryonic fibroblasts (STO) cell line	Hypotonic lysis buffer, isolation of cell membranes by centrifugation	(1) PPS Silent (2) AALS I (3) AALS II (4) CALS I (5) CALS II (6) ProteaseMAX (7) RapiGest	TCEP/IAA	trypsin	degradation under acidic conditions	RP, C18-AQ, water-ACN grad., 0.1% (*v*/*v*) FA	ESI-LIT(LTQ)-MS	[148]
53	A375 cell line	Detergent-containing lysis buffer, sonication	(1) ProteaseMAX (2) SDC	DTT/IAA	trypsin	(1) PPS Silent: degradation under acidic conditions (2) SDC: acid precipitation	RP, C18 PepMap, water-ACN grad., 0.1% (*v*/*v*) FA	ESI-Q-Orbitrap-MS	[119]
54	HEK293 kidney cell line	Detergent-containing lysis buffer, sonication	(1) SDC (2) PPS Silent (3) Invitrosol (4) RapiGest	DTT/IAA	trypsin	(1) PPS Silent and RapiGest: degradation under acidic conditions (2) SDC: acid precipitation (3) Invitrosol: none	RP, C18, water-ACN grad., 0.1% (*v*/*v*) FA	ESI-Q-Orbitrap-MS	[150]
55	*Escherichia coli*	Detergent-containing lysis buffer	SDS	DTT/IAA	trypsin	(1) FASP (2) Protein precipitation in 80% acetone (3) Protein precipitation in TCA with acetone wash (4) KDS precipitation (5) Pierce detergent removal spin columns (6) SCX (7) in-gel digestion	RP, C18, water-ACN grad., 0.1% (*v*/*v*) FA	ESI-QqQ-MS	[149]
56	Mouse pancreatic islets	Centrifugation, detergent-containing lysis buffer, sonication	(1) urea (2) SDS (3) SDC (4) NP-40 (5) RapiGest.	DTT/IAA	trypsin	FASP	RP, C18, water-ACN grad., 0.1% (*v*/*v*) FA	ESI-Q-Orbitrap-MS	[153]
57	*Solanum esculentum* L. (tomato) roots	Detergent-based extraction	(1) PPS Silent (2) RapiGest (3) GdnCl	TCEP/IAA	trypsin	PPS Silent and RapiGest: degradation under acidic conditions	RP, BEH C18, water-ACN grad., 0.1% (*v*/*v*) FA	ESI-QqTOF-MS	[155]

AALS, anionic acid labile surfactant; ACN, acetonitrile; BSA, bovine serum albumin; CAA, chloroacetamide; CHAPS, 3-[3-(cholamidopropyl) dimethyl-ammonio]-1-propanesulfonate; DCA, deoxycholic acid; DTT, dithiothreitol; ESI, electrospray ionization; FA, formic acid; FASP, filter-aided sample preparation; FT-ICR, Fourier transform-ion cyclotron resonance; ; GdnHCl, guanidinium chloride; HCIT, high-capacity ion trap; IAA, iodoacetamide; IT, ion trap; KDS, potassium dodecyl sulfate; LIT—linear ion trap; MALDI-TOF, matrix assisted laser desorption/ionization—time of flight; MS, mass spectrometry; nano-scaled liquid chromatography; Q, quadrupole mass analyzer; QqTOF, quadrupole-time of flight; RP, reversed phase; SCX, strong-cation exchange; SDC, sodium deoxycholate; SDS, sodium dodecyl sulfate; SP3, single-pot solid-phase-enhanced sample preparation; S-Trap, Suspension trapping; TCA, trichloroacetic acid; TCEP, tris-(2-carboxyethyl)-phosphine.
